# Downregulation of the anaphase-promoting complex (APC)7 in invasive ductal carcinomas of the breast and its clinicopathologic relationships

**DOI:** 10.1186/bcr978

**Published:** 2005-01-14

**Authors:** Kwang-Hwa Park, Sung-E Choi, Minseob Eom, Yup Kang

**Affiliations:** 1 Department of Pathology, Yonsei University, Wonju, Korea; 2 Institute for Medical Science, Ajou University School of Medicine, Suwon, Korea

**Keywords:** anaphase-promoting complex, aneuploidy, breast cancer, histologic grade, Ki-67

## Abstract

**Introduction:**

The anaphase-promoting complex (APC) is a multiprotein complex with E3 ubiquitin ligase activity, which is required for the ubiquitination of securin and cyclin-B. Moreover, the mitotic spindle checkpoint is activated if APC activation is prevented. In addition, several APC-targeting molecules such as securin, polo-like kinase, aurora kinase, and SnoN have been reported to be oncogenes. Therefore, dysregulation of APC may be associated with tumorigenesis. However, the clinical significance and the involvement of APC in tumorigenesis have not been investigated.

**Methods:**

The expression of APC7 was immunohistochemically investigated in 108 invasive ductal carcinomas of the breast and its relationship with clinicopathologic parameters was examined. The expression of APC7 was defined as positive when the summed scores of staining intensities (0 to 3+) and stained proportions (0 to 3+) exceeded 3+.

**Results:**

Positive APC7 expression was less frequent than its negative expression when histologic (*P *= 0.009) or nuclear grade (*P *= 0.009), or mitotic number (*P *= 0.0016) was elevated. The frequency of APC7 negative expression was higher in high Ki-67 or aneuploid groups than in low Ki-67 or diploid groups.

**Conclusion:**

These data show that loss of APC7 expression is more common in breast carcinoma cases with poor prognostic parameters or malignant characteristics. They therefore suggest that dysregulation of APC activity, possibly through downregulation of APC7, may be associated with tumorigenesis in breast cancer.

## Introduction

The anaphase-promoting complex (APC) is an E3 ubiquitin ligase that controls mitotic progression [1,2]. APC is a polymeric protein complex composed of at least 11 subunits, which contains tetratricopeptide repeat proteins (APC3, 5, 6, 7, and 8), a cullin homolog (APC2), and a ring-H2 finger domain (APC11). APC requires two WD40 repeat-containing coactivators, Cdc20 and Cdh1, to recruit and select various substrates at different stages of the cell cycle, and it was recently suggested that APC3 and APC7 interact with these APC activators [3]. APC promotes metaphase/anaphase transition by ubiquitizing and degrading securin, an inhibitor of separase that participates in the degradation of the chromatic cohesion complex. APC also ubiquitinates cyclin-B and accelerates its degradation during the late mitotic to the G_1 _phase, which results in mitotic exit. In addition, APC is known to target various cell cycle regulatory molecules, including spindle-associated protein, DNA replication inhibitors, and mitotic kinases.

Several molecules targeted by APC have been reported to promote transformation. Pituitary tumor-transforming gene (PTTG), a vertebrate analog of securin, has been reported to be an oncogene [4], and cancerous tissues from patients with leukemia, lymphoma, or testicular, ovarian, breast, or pituitary cancer were found to over-express PTTG [5-7]. It was further reported that the constitutive expression of polo-like kinase (PLK), a serine/threonine kinase that is involved in spindle formation, centrosome cycles, and chromosome segregation [8], may induce tumor formation [9]. Several reports have suggested a role for PLK in the progression and/or malignancy of human cancers, such as glioma, and endometrial carcinoma, breast, ovarian, and esophageal carcinoma [10-13]. Aurora kinase, another serine/threonine kinase that is involved in chromosome segregation and centrosome maturation [14], has also been reported to be amplified in bladder, gastric, breast, and colorectal cancers [15-18] and to have the ability to transform NIH3T3 cells [19]. Recently, SnoN, a negative regulator of Smad that is involved in the transforming growth factor-β signaling pathway, was shown to be a target molecule for the APC [20,21] and to have transforming potential [22]. It was also found that SnoN is amplified in stomach, thyroid, and lung carcinoma and lymphoma [23]. APC-regulating molecules have also been reported to be involved in transformation. RASSF-1A and Mad2, which inhibit APC activity, were reported to be tumor suppressors [24,25].

Chromosome instability is believed to contribute to malignant transformation because the majority of malignant human cancers exhibit chromosomal gain or loss [26] and because mitotic defects including chromosome aberrations are frequently found in malignant cancers [27-29]. Because of the roles played by APC in mitotic cell cycle progression, the timely activation of APC is thought to be important for maintaining accurate chromosome separation. In addition, a report indicating that the mitotic spindle checkpoint was reached by preventing APC activation [30] suggests that the dysregulation of APC may give rise to abnormal chromosome segregation, resulting in aneuploidy. The recent finding that APC5 deficiency in *Drosophila *is accompanied by a mitotic defect, which included aneuploidy, suggests a role for APC in the maintenance of chromosome stability [31]. It can therefore be hypothesized that the abnormal regulation of APC may be involved in malignant transformation through chromosome instability.

However, it is not known whether the abnormal regulation of APC, possibly through genomic mutation or the modulation of APC components, is related to tumorigenesis. Furthermore, whether dysregulation of APC is related to clinical parameters in various human cancers is yet to be determined. Thus, we investigated immunohistochemically the levels of APC7 in various cancer tissues and found weak APC7 expression in high-grade ductal carcinomas of breast. Therefore, we were encouraged to investigate the expression of APC7 in 108 breast carcinomas and to examine the relationship between the expression of APC7 and clinicopathologic parameters.

## Methods

### Production of polyclonal antibodies against APC7

Polyclonal antibodies against mouse APC7 were raised in a NZW rabbit by immunization with recombinant APC7 protein. Briefly, recombinant mouse APC7 proteins were produced in *Escherichia coli *using a pET32 expression vector system (Novagen, Madison, WI, USA). The resulting 6× histidine-tagged APC7 proteins were purified by Ni-NTA affinity chromatography (Qiagen, Hilden, Germany). A NZW rabbit was then immunized with the purified APC7 protein and boosted twice. Blood was collected from the auricular artery, and serum was prepared by clotting and differential centrifugal separation (10,000 *g *for 10 min). APC7-specific antibodies were further purified by binding serum to APC7-coupled nitrocellulose and eluting with 100 mmol/l glycine–HCl buffer (pH 2.5).

### Immunoblotting and immunoprecipitation

Protein extracts were prepared by solubilizing cells in RIPA buffer (150 mmol/l NaCl, 1% NP40, 0.5% deoxycholate, 0.1% sodium dodecylsulfate, 50 mmol/l Tris.Cl, pH 7.5, protease inhibitors) and differential centrifugation (10,000 *g *for 10 min). Of the protein fractions obtained, 30 μg was resolved by 12% SDS-PAGE, and then the separated proteins were electrotransferred onto Immobilon membranes (Millipore, Bedford, MA, USA). After preblocking these membranes with 5% skimmed milk, they were treated with anti-mouse APC7 or human APC7 (Santa Cruz Biotechnology, Santa Cruz, CA, USA) antibodies as primary antibody and horseradish peroxidase-conjugated anti-rabbit antibodies as secondary antibody. Immunoreactive bands were developed using an electrogenerated chemiluminescence system (Amersham Pharmacia Biotech, Uppsala, Sweden). Immunoprecipitation was carried out with anti-human APC3 antibodies (Transduction Laboratory, San Diego, CA, USA) or anti-mouse APC7 antibodies. Subconfluent cells were collected and then lysed by incubation on ice for 15 min in EBC buffer (0.5% Nonidet P-40, 40 mmol/l Tris.HCl, pH 8.0, 120 mmol/l NaCl, 10 μg/ml aprotinin, 10 μg/ml leupeptin, 10 μg/ml PMSF). Cell lysates (1 mg) collected by differential centrifugation (12,000 *g *for 15 min) were mixed with 1 μg anti-human APC3 antibodies or anti-APC7 antibodies, and these mixtures were then further incubated for 1 hour at 4°C. Immune complexes were collected by incubating with 30 μl of 50% protein A-sepharose slurry for 1 hour and centrifugation (10,000 *g *for 15 s). After washing three times with ice-cold EBC buffer, the pellets were suspended in 2× loading buffer (125 mmol/l Tris.HCl, pH 6.8, 4% SDS, 20% glycerol, 14.4 mmol/l 2-mercaptoethanol) and boiled for 5 min. Immunoblotting was carried out with anti-human APC3, anti-human APC6 (Santa Cruz Biotechnology), or anti-mouse APC7 antibodies.

### Tissue samples

Paraffin wax embedded blocks containing breast tumor tissues resected from 108 patients diagnosed as having invasive ductal carcinoma of breast at Wonju Christian Hospital (Wonju, Korea) between January 1996 and May 2001 were used in this study. Patient ages ranged from 24 to 81 years (mean 47.5 years). All procedures were performed in accordance with our hospital's ethical guidelines, and approval for the study was granted by the university hospital's ethics committee. All patients provided informed consent.

### Pathologic examination

Hematoxylin–eosin stained slides were reviewed, and histologic grade was determined in terms of tubule formation, nuclear pleomorphism and mitosis, using the criteria described by Bloom and Richardson [32]. Tumor size, lymphatic metastasis, and clinical stage were determined according to the American Joint Committee on Cancer criteria [33].

### Immunohistochemistry and evaluation

Specimens were fixed in 10% buffered formaldehyde and embedded in paraffin using routine methods. Sections 5 μm thick were placed on silane-coated glass slides, dried at 50°C for 2 hours, deparaffinized in xylene, rehydrated in graded ethanol, and then washed in distilled water. To retrieve antigenicity, the sections were dipped in citrate buffer (10 mmol/l, pH 6.0) in a tender cooker (Nordic Ware, Minneapolis, MN, USA) and then warmed for 15 min in a microwave oven. Endogenous peroxidase activity was blocked by pretreating with 0.3% hydrogen peroxide for 10 min. After washing with 50 mmol/l Tris buffer (pH 7.5), primary antibodies, namely anti-mouse APC7, human APC7, human Ki-67 (DAKO, Copenhagen, Denmark), or estrogen receptor (ER) antibodies (Novocastra, Newcastle, UK), were applied overnight at a dilution of 1:50 or 1:100. The sections were then further incubated for 20 min in a 1:50 dilution of biotinylated goat anti-rabbit or rabbit anti-mouse antibody (DAKO) as secondary antibody. Color was developed by incubating with streptavidin peroxidase (DAKO) for 20 min and staining with 3-amino-9-ethylcarazole. Counter-staining was carried out with hematoxylin before mounting. To obtain relevant staining equivalence of APC7 in different carcinoma tissues, an unstained tissue sample and a strongly stained tissue sample were used as negative and positive control, respectively. Whenever a staining procedure was performed, negative and positive control tissues were simultaneously stained with new battery of tissues and then the control tissues were used as a staining reference.

All slides were examined by three pathologists and scores were determined by consensus. The immunohistochemical intensity of APC7 was awarded an 'intensity' score of 0 to 3+, with 0 represented an unstained nucleus and 3+ the strongest staining intensity. The 'proportion' score represented the estimated percentage of stained cells as a fraction of all tumor cells in the microscopic field (i.e. 0 = 0%, 1+ = 0–25%, 2+ = 25–50%, and 3+ = >50%). Because true negative expression with a staining intensity of 0 was very rare (six cases/108 tissue samples), and therefore the statistical significance between APC7 expression and clinicopathologic parameters could not obtained, we included a weak APC7 expression group (34 cases/108 tissue samples) with staining intensity of 1 and proportion score of 1 in the negative group. Therefore, a summed intensity and proportion score of ≥ 3+ was defined as positive APC7 expression whereas a score of ≤ 2+ was defined as negative. The Ki-67 labeling index was defined as the percentage of positively stained cells in five to seven high power fields (×400). At least 1000 cells per field were counted. Nuclear ER staining was also examined at ×400 and compared with a strong positive control. ER staining intensity was designated weak, moderate, or strong. Positive reactivity was defined when the proportion of cells exhibiting moderate to strong staining exceeded 10%.

### DNA analysis

Two 50-μm sections were cut from each paraffin block, deparaffinized in xylene, rehydrated in a descending ethanol series, and then washed in phosphate-buffered saline. The sections were then placed in 10 mmol/l citrate solution (pH 6.0) and incubated for 2 hours at 80°C. After cooling, 1 mg/ml pepsin in 0.1 N HCl was added and the sections were digested for 30 min. The resulting suspension (2 × 10^6 ^nuclei) was filtered through 50 μ-mesh and further suspended in 500 μl of 1% bovine serum albumin solution. DNAs were stained using a Cycle TEST PLUS DNA Reagent Kit™ (Becton Dickinson, Ontario, Canada). Stained cells were analyzed using a FACscan (Becton Dickinson, San Jose, CA, USA) and the fraction of aneuploid cells was calculated using Cell Fit software (Becton Dickinson).

### Statistical analysis

Statistical analysis was performed using the SPSS ver. 10.0 program (SPSS Inc., Chicago, IL, USA). The association between APC7 expression and clinicopathologic parameters was analyzed using χ^2 ^tests. *P *≤ 0.05 was considered statistically significant.

## Results

### Characterization of polyclonal antibodies against APC7

**Figure 1:**
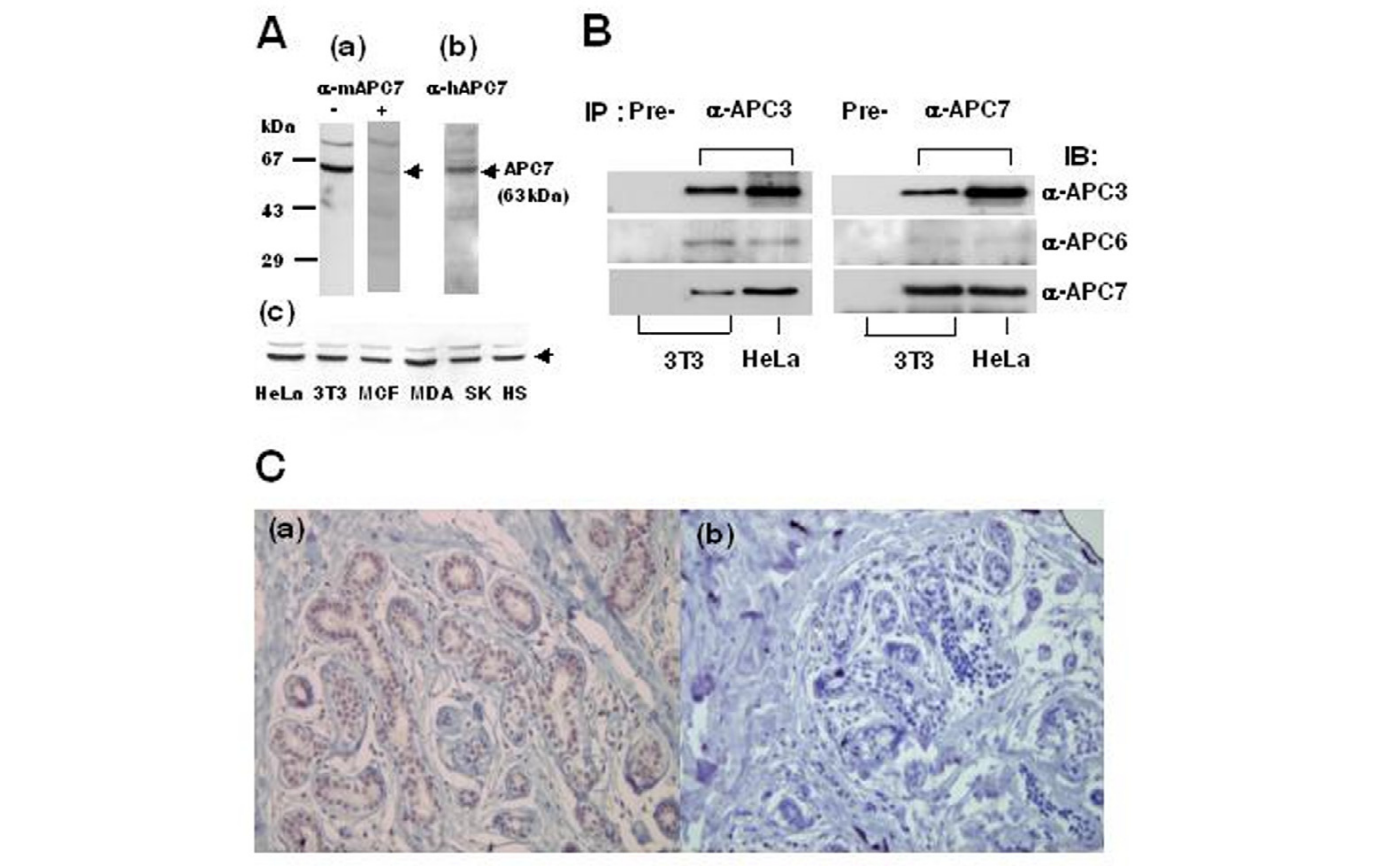
Recognition of anaphase-promoting complex (APC)7 antigen with anti-mouse APC7 antibodies. **(A) **Panel a shows immunoblotting of human MCF-7 breast carcinoma cell extracts with affinity-purified anti-mouse APC7 polyclonal antibodies before (-) and after (+) prebinding APC7 antibodies to recombinant mouse APC7-coupled nitrocellulose. Panel b shows immunoblotting with human APC7 polyclonal antibodies (Santa Cruz; sc-20987). Panel c shows immunoblotting of human Hela cervical carcinoma, mouse NIH 3T3 fibroblast, and human breast carcinomas (MCF, MCF-7; MDA, MDA-MB-231; SK, SK-BR-3; HS, HS 578T) with purified mouse APC7 antibodies. Cell extracts were resolved on 12% SDS-PAGE and the separated proteins were electrotransferred onto Immobilon membranes (Millipore). The membranes were then treated with anti-APC7 antibodies and horseradish peroxidase-conjugated anti-rabbit IgG antibodies as primary and secondary antibodies, respectively. Immune reactive bands were developed using an electrogenerated chemiluminescence (ECL) system. **(B) **Immunoprecipitation and immunoblotting of mouse 3T3 and human Hela cell extracts with anti-human APC3 (CDC27; Transduction Laboratory) or purified anti-mouse APC7 antibodies. After mixing cytosolic extracts (1 mg) with pre-immune rabbit sera (2 μl), anti-APC3 (1 μg), or anti-mouse APC7 antibodies (1 μg), precipitates were fractionated on 12% SDS-PAGE. After electrotransfer, immunoblotting was performed with anti-APC3, anti-human APC6 (Santa Cruz Biotech), or anti-mouse APC7 antibodies as primary antibodies. Peroxidase-conjugated anti-mouse, anti-goat, or anti-rabbit IgG antibodies were bound as secondary antibodies and developed using the ECL system. **(C) **Immunohistochemistry of normal breast tissues with purified anti-mouse APC7 antibodies before (panel a) and after (panel b) prebinding APC7 antibodies to recombinant mouse APC7-coupled nitrocellulose (original magnification ×200). After binding the purified anti-mouse APC7 antibodies and the biotinylated goat anti-rabbit antibodies as primary and secondary antibodies, respectively, a streptavidin-peroxidase kit (DAKO) was used to detect APC7 antigen. Staining and counter-staining were then performed using 3-amino-9-ethylcarazole and hematoxylin, respectively.

In this study we isolated a novel gene (GenBank Accession Number: AF076607) and identified it as the mouse APC7 gene (GenBank Accession Number: BC006635). It was found to have 97.7% homology with its human counterpart (GenBank Accession Number: AF191340). Polyclonal antibodies were raised by immunizing a NZW rabbit with recombinant mouse APC7 proteins (amino acids 88–565), and the APC7-specific antibodies so obtained were then purified by affinity binding to APC7-coupled nitrocellulose. Immunoblotting analysis of MCF-7 human breast carcinoma extracts showed that these purified antibodies and human APC7 antibodies (sc-20987; Santa Cruz) recognized a distinct 63-kDa band, and that this immune reactivity was APC7-specific (Fig. 1A, panels a and b). The antibodies recognized same sized antigens from mouse and human cells (Fig. 1A, panel c). Moreover, APC7 antibodies precipitated both APC3 and APC6 components in mouse and human derived cells, whereas human CDC27 (APC3) antibodies precipitated APC6 and APC7 components (Fig. 1B), thus demonstrating that the antigen recognized by our purified antibody is the APC7 component of the APC. Immunohistochemical studies on the paraffin-embedded sections of normal breast tissues showed that the purified APC7 antibodies recognized antigens located in the nucleus (Fig. 1C, panel a), which is in accordance with the finding that most APC antigens are localized in nucleus during the interphase [33]. Immune reactivity, as shown by immunohistochemistry, was also APC7 specific (Fig. 1C, panel b).

### Expression of APC7 in various human tissues

To search for differentially expressed APC7 in normal and cancerous tissues, we performed immunohistochemical analyses with the purified mouse APC7 antibodies using tissue array slides containing 50 normal or 50 tumor tissue cores. We compared the APC7 expressions of the cores by assessing the averaged staining intensities (0 to 3+). Staining of ≥ 2+ was defined as positive expression and of ≤ 1+ as negative expression. Table 1 lists the APC7 expression of 17 normal and 22 tumor tissues with multiple cores. Positive staining was observed in rapid growing normal epithelial tissues. In contrast, slow growing but more differentiated tissues such as skeletal muscle, adipocytes, spinal cord, brain, and basal stromal tissues near epithelial cells exhibited no or weak immune reactivity to APC7. In addition, slowly growing tumors such as chondrosarcomas, lipomas, low-grade urothelial carcinomas, and renal cell carcinomas tended to show weak reactivity to APC7, whereas most tumor tissues with high proliferation rate were positive. Interestingly, some ductal carcinomas of the breast with an undifferentiated high histologic grade exhibited weak reactivity to APC7.

### Relationship between APC7 expression and clinicopathologic parameters in breast carcinomas

**Figure 2:**
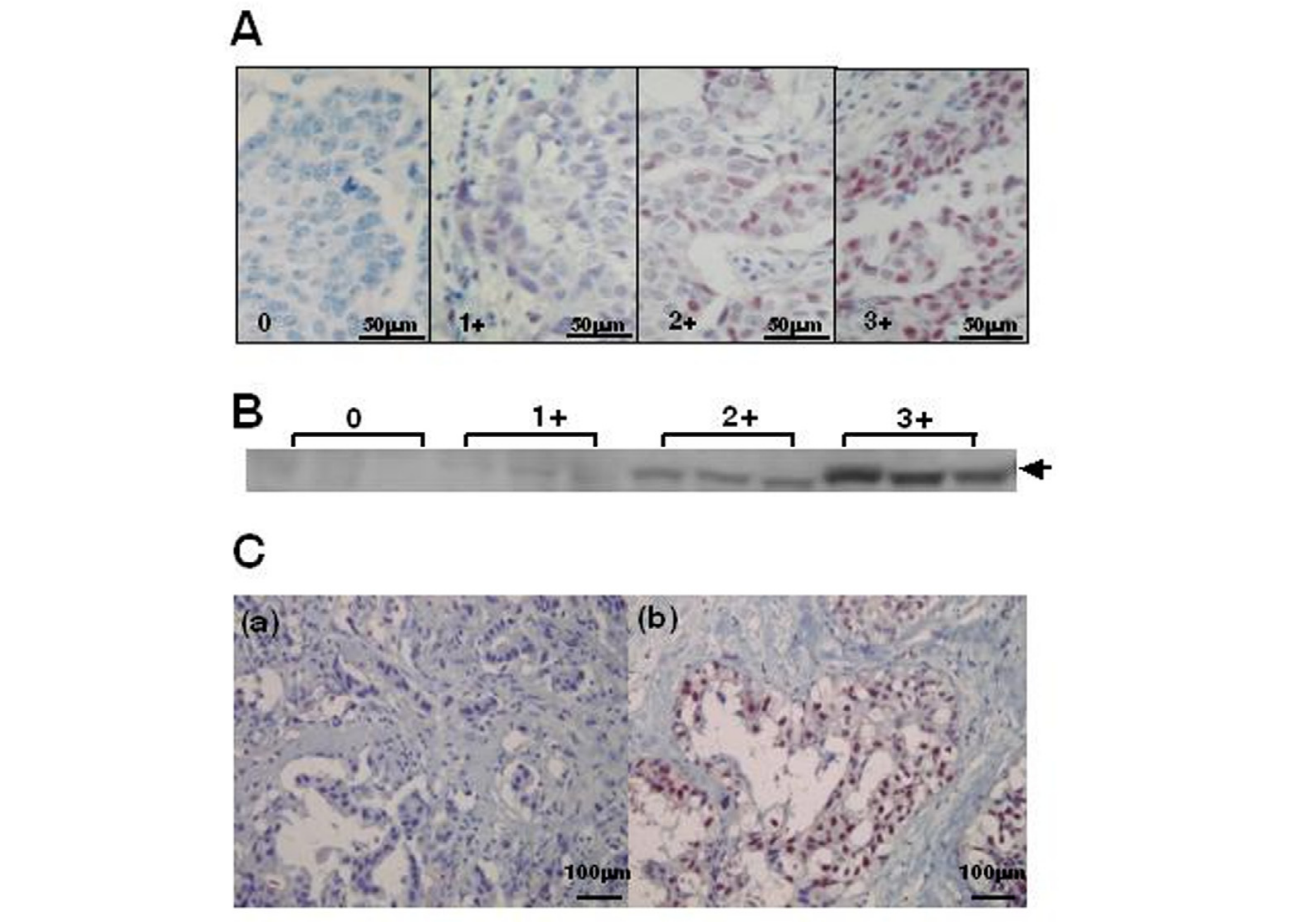
Representative immunohistochemical and immunoblotting analysis of anaphase-promoting complex (APC)7 in invasive ductal carcinomas of the breast. **(A) **Invasive ductal carcinomas stained by APC7 antibody with intensity scores of 0 to 3+ (original magnification, ×400). After binding with anti-mouse APC7 antibodies as primary antibodies and with biotinylated anti-rabbit antibodies as secondary antibodies, streptavidin peroxidase was bound to the biotinylated antibodies. Staining was conducted with 3-amino-9-ethylcarazole and hematoxylin. **(B) **Immunoblotting of three breast tissues with APC7 intensities ranging from 0 to 3+. Immunoblotting was carried out using affinity-purified APC7 antibodies and peroxidase-conjugated anti-rabbit IgG antibodies as primary and secondary antibodies, respectively. Electrogenerated chemiluminescence was used to detect positive bands. **(C) **Representative negative (panel a) and positive (panel b) APC7 immunostaining of invasive ductal breast carcinoma with histologic grades of 3 and 2, and staining intensities of 0 and 3+, respectively. Immunohistochemical staining was also performed using a streptavidin peroxidase kit.

To determine whether the loss of APC7 expression is related to tumorigenesis in breast cancer, we scrutinized the expression level of APC7 immunohistochemically in 108 invasive ductal carcinomas of the breast and then searched for correlations with clinocopathologic parameters. Figure 2A shows the representative features of APC7 staining scores of 0 to 3+, and Fig. 2B shows the immunoblotting results for three representative tissues with different intensity scores. These data show that APC7 staining intensity was proportional to band intensity by immunoblotting, demonstrating that immunohistochemical staining intensities represent APC7 expression. Typical negative and positive APC7 expressions in breast carcinoma are shown in Fig. 2C panels a and b. The ratios of APC7-positive to APC-negative expression and their relationships with various clinicopathologic parameters are summarized in Table 2. Of breast carcinomas, 63% exhibited positive APC7 expression and 37% were negative. APC7 expression did not correlate with tumor size (*P *= 0.8180) or metastasis (*P *= 0.9703). No statistical significance was found between clinical stage (0.2798) and APC7 expression, or between ER expression (0.1031) and APC7 expression. Nevertheless, the frequency of positive APC7 expression tended to be lower in clinical stage III (58.3%) than in stage I (78.9%) tumors, and in patients who were ER negative (52.9%) than in those who were ER positive (70.6%). In contrast, negative APC7 expression was highest in stage III tumors and in those who were ER negative.

On the other hand, the negative expression of APC7 was positively correlated with higher histologic grade (*P *= 0.001), nuclear grade (*P *= 0.0090), mitotic number (*P *= 0.0016), Ki-67 index (*P *= 0.0078), and aneuploidy (*P *= 0.0095). Moreover, APC7 expression was more frequent in those with a low histologic grade (84.8%) than in those with a high grade (35.0%). Because histologic grade is determined by nuclear pleomorphism, mitotic number, and tubule formation, those with a high nuclear grade and a high mitotic number also exhibited a similar negative correlation with APC7 expression. The frequency of positive APC7 expression was lower in the high Ki-67 group than in the low Ki-67 group. About 82% (77/94) of tissue samples were classified as aneuploid. Nearly half of the aneuploid group exhibited a low level of APC7 expression (42.9%), whereas most of the diploid group showed positive APC7 expression (94.1%), indicating that breast carcinomas with normal ploidy express higher levels of APC7.

### Immunoblotting analysis of APC expression in breast carcinomas

To determine whether the expression of the APC7 component is exclusively modulated in breast carcinoma, we investigated the expression levels of other APC components. We first performed immunohistochemic analysis using anti-human APC3 antibodies or anti-human APC6 antibodies. However, we could not obtain meaningful data because nuclei in all breast carcinoma tissues were strongly stained by these antibodies, probably because of nonspecific cross-reactivity. Next, we compared the expression levels of APC3 and APC6 components by immunoblotting. However, immunoblotting analysis with anti-human APC6 antibodies also failed to exhibit a distinct band in tumor tissues because of the weak immune reactivity and the nonspecific reactivity of the APC6 antibody. Thus, we were able to obtain expression data on APC3 in various breast carcinoma tissues, in conjunction with APC7 expression.

**Figure 3:**
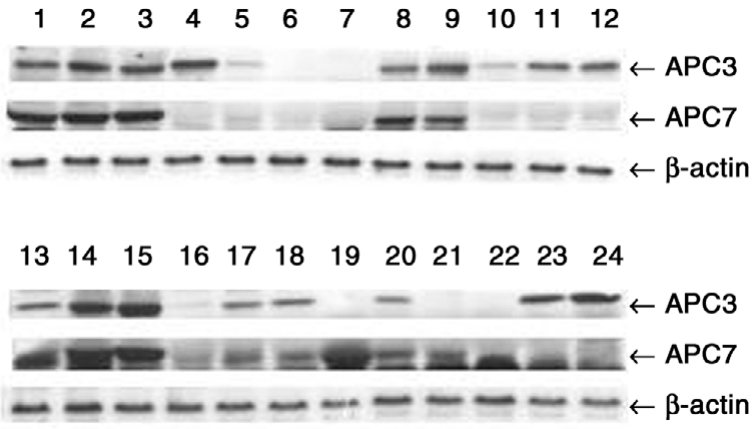
Immunoblotting analysis of breast carcinoma using anti-anaphase-promoting complex (APC)3 and APC7 antibodies. Protein extracts were prepared from 24 ductal breast carcinoma tissues by homogenizing tissues in EBC buffer for 1 min and centrifuging (12,000 *g *for 15 min). Of protein from each tissue, 30 μg was fractionated by 12% SDS-PAGE and transferred to Immobilon membranes. Immunoblotting analysis was also carried out using anti-APC3 or APC7 antibodies as primary antibodies, and horseradish peroxidase-conjugated anti-mouse or anti-rabbit IgG antibodies as secondary antibodies, and bands were then detected by electrogenerated chemiluminescence.

Figure 3 shows immunoblotting results for APC3 and APC7 in 24 representative breast carcinoma tissues. The expression levels of APC3 and APC7 in these tissues was variable, which might have been due to variable APC expression in these tissues. Some tissues (lanes 1, 2, 3, 8, 9, 13, 14, and 15) exhibited relatively high levels of expression of both APC3 and APC7, whereas other tissues (lanes 6, 7, 16, and 22) showed no expression of APC3 and APC7. These data suggest that the expression levels of APC3 and APC7 are simultaneously regulated in some breast carcinoma tissues. On the other hand, several carcinomas (lanes 4, 11, 12, 23, and 24) showed relatively high APC3 expression but low APC7, suggesting that selective downregulation of APC7 is unique to some breast carcinomas.

## Discussion

This work was undertaken to determine whether the expressional modulation of APC7 is related to tumorigenesis in human cancers. We first used immunohistochemistry to investigate the expression of APC7 in tissue array slides mounted with various cancer tissues, and we observed strong immune reactivity to APC7 in the most rapidly growing tumor tissues. However, some breast cancer tissues with a high histologic grade exhibited weak immune reactivity to APC7. Therefore, we scrutinized APC7 expression in 108 invasive ductal carcinomas of the breast and compared these findings with clinicopathologic parameters. Although positive immune reactivity to APC7 was observed in more than 60% of breast carcinomas, negative APC7 expression was frequently observed in breast carcinomas with more aggressive characteristics (i.e. a higher histologic grade, a higher nuclear grade, a higher proliferation rate, or aneuploidy). These findings suggest a possible association between the expression of APC7 and breast cancer tumorigenesis.

Most components of APC have been reported to be expressed in growing tissues at fairly constant levels [34]. On the other hand, Gieffers and coworkers [35] reported that components of APC (i.e. APC2, APC3, and APC7) are expressed in postmitotic adult brain tissue. However, it is not known how the expressions of APC components are modulated according to growth or cell differentiation. We observed twofold APC7 modulation in mouse NIH3T3 cells according to cell cycle (data not shown). In the present study, immunohistochemical studies using normal and cancer tissue arrays showed that APC7 is highly expressed in most proliferating cells. Strong immunoreactivity to APC7 was restricted to normal epithelial tissues and proliferating cancer tissues, whereas low APC7 immunoreactivity was observed in slow growing and differentiated tissues, such as adipocytes, hepatocytes, muscle cells, brain, and spinal cord, and in slowly growing tumor tissues such as lipoma, pleomorphic adenoma of the salivary gland, adenoid cystic carcinoma, chondrosarcoma, low-grade urothelial carcinoma, and renal cell carcinoma.

Interestingly, we found a negative correlation between APC7 expression and some high-grade breast carcinoma tissues, and especially in those with aneuploidy. This negative correlation seems to be unique to some malignant breast carcinomas because we did not observe significant loss of APC7 expression in other aggressive carcinomas. In fact, we further investigated APC7 expression in two representative carcinomas, namely lung and renal carcinomas (i.e. rapid and slow growing carcinomas, respectively; data not shown), and obtained the same result as that obtained using the tissue array. All rapidly growing carcinoma tissues examined showed positive APC7 expression, whereas over 90% of slow growing renal carcinomas showed negative APC7 expression. Reports that the level of APC1 expression in breast cancer tissue is lower than that in other cancer tissues, and that expressed sequence tags of APC7 are reduced in breast tissues [36] support our observation that the loss of APC7 expression seems to be restricted in some high-grade breast carcinomas. Immunoblotting analysis showed that the expression of APC7 was variable in different breast carcinomas, which appears to be due to differences in expression of APC7 between individual breast carcinomas. However, the possibility of epithelial cell contamination during tissue extraction cannot be excluded. In several breast carcinomas, the loss of APC7 expression was accompanied by the loss of another APC component, such as APC3, but other tissues exhibited selective APC7 downregulation. These observations demonstrate that a unique loss of APC7 expression occurs in some breast carcinomas, and suggest that regulation of APC components in breast carcinomas is heterogeneous. Our data agree with reports that the expressional patterns of APC components are not simultaneously modulated [37] and that APC components can be individually modulated by environmental stimuli [38].

It has been reported that chromosome instability through abnormal mitotic progression plays a critical role in tumor malignancy [38,39]. Therefore, the dysregulation of APC activation, which probably perturbs mitotic progression, may affect malignant transformation or tumor progression. Moreover, the finding that APC is required for the G_2 _and mitotic checkpoints suggests that malignant transformation can be caused by chromosome instability through the dysregulation of APC activation [40]. Recently, Wang and coworkers [41] reported a genetic alteration in APC6 and APC8 in human colon cancer cells, and suggested their involvement in colon carcinogensis. Aneuploidy is frequently observed in breast cancer tissues [42], and APC target molecules such as PTTG, PLK, and aurora kinase are often upregulated in the same tissues [7,12,17], supporting the notion that dysregulation of APC may play a role in the tumorigenesis of breast cancer.

Our data concerning the negative correlation between APC7 expression and a high histologic grade with aneuploidy supports a possible linkage between the downregulation of APC7 and malignant transformation in breast cancer. As several papers have reported lethality induced by the loss of APC components [43,44], our observation raises the question as to how the cell cycle can progress in the absence of APC7. Although defects in the spindle checkpoint could elicit cell death, cancer cells in conjunction with p53 mutation could override the mitotic checkpoint and the cell lethality elicited by abnormal mitosis [41]. In this situation, it is believed that an abnormality in APC regulation could induce unscheduled mitotic progression [31]. One interesting observation was that the cell cycle of mutant APC8-harboring cells progressed, even with a disturbed pattern [45]. A recent report that the expression of cyclin-B_1 _in breast cancer cells was sustained in the G_1 _phase suggests that the cell cycle can progress in the presence of an abnormal mitotic cell cycle machinery [46]. Therefore, we hypothesize that cells with a functional APC defect can drive cell cycle progression in a situation in which cyclin-B has accumulated. However, the mechanism that allows breast cancer cells to progress through mitosis with functionally defective APC remains unknown.

We did not determine whether APC7 expression is correlated with disease-free survival because most of the patients had recently been diagnosed. However, our data demonstrate that the downregulation of APC7 is more common in those who exhibit markers of poor prognosis. A number of parameters, namely lymph node metastases, tumor size, histologic grade, ER and progesterone receptor expression, lymphovascular invasion, proliferation rate, DNA content, and expression of oncogenes, have been reported to influence the prognosis of women with breast cancer [47]. On the other hand, in the present study poor prognostic indications such as high histologic grade, high proliferation rate, and aneuploidy were found to be related to downregulation of APC7, which suggests that breast cancer patients exhibiting weak APC7 expression would have poor survival. Yoshimoto and coworkers [48] reported that histologic grade and clinical stage, including lymph node metastasis, are important prognostic markers in breast cancer patients, which supports the notion that the downregulation of APC7 can be a viable prognostic marker. An earlier report that the Ki-67 index is related to histologic grade also supports the prognostic significance of the negative correlation between APC7 and proliferation [49].

## Conclusion

We found that downregulation of APC7 in breast carcinoma is more common in those with high histologic grade and high proliferation rate, and in those showing aneuploidy. Therefore, downregulation of APC7 may be a marker of a poor prognosis and may contribute to breast cancer tumorigenesis via chromosome instability and/or accelerated oncogenic signaling.

## Abbreviations

APC = the anaphase-promoting complex; ER = estrogen receptor; PLK = polo-like kinase; PTTG = pituitary tumor transforming gene.

## Competing interests

The author(s) declare that they have no competing interests.

## Authors' contributions

KP carried out immunohistological studies and statistical analysis. SC produced mouse APC7 proteins and prepared APC7 antibodies. She also performed immunoblotting studies. ME participated in pathologic and immunohistologic studies. YK designed and coordinated this research and prepared the manuscript.

## Figures and Tables

**Table 1:** Expression of APC7 in various human tissues

Expression of APC7^a^	Tissue type	
	
	Normal tissue	Tumor tissue
Negative	Adipocytes, brain, hepatocytes, skeletal muscle cells, spinal cord	Lipoma, pleomorphic adenoma of salivary gland, low grade urothelial carcinoma, chondrosarcoma, adenoid cystic carcinoma, renal cell carcinoma, high grade ductal carcinoma of breast
Positive	Basal cells of epidermis, bronchial epithelium, ductal cells of breast, ductal cells of pancreas, ductal cells of salivary glands, endometrial glands, kidney epithelium, gastric mucosa, prostate glands and ducts, urothelial epithelium, fibroblasts, germinal center cells	Adenocarcinoma of colon, adenocarcinoma of endometrium, adenocarcinoma of pancreas, adenocarcinoma of prostate, adenocarcinoma of stomach, ductal carcinoma of pancreas, hepatocellular carcinoma, high grade urothelial carcinoma, papillary serous carcinoma of ovary, squamous cell carcinoma of esophagus, squamous carcinoma of cervix, leiomyosarcoma of uterus, malignant lymphoma, melanoma, seminoma of testis

^a^Immunohistochemical data from tissue arrays (Tissue-Array Co., Seoul, Korea) stained with anti-anaphase-promoting complex (APC)7 antibodies. Tissue-array slides mounted with 50 normal or 50 tumor cores contained 17 normal or 22 tumor tissues, respectively, in triplicate or duplicate. Negative APC7 expression is represented by staining intensities of 0 or 1+, whereas positive expression is represented by staining intensities of 2+ or 3+, as designated in Fig. 2A. Average staining intensities of several cores were used to determine tissue expression.

**Table 2:** Relationship between APC7 expression and various clinicopathological parameters in invasive ductal carcinomas of the breast

Characteristics	All cases	APC7 positive	APC7 negative	*P*^a ^(significance)
All carcinomas	108 (100%)	68 (63.0%)	40 (37.0%)	
Tumor size				0.8180 (NS^b^)
Small (<5 cm)	81 (100%)	51 (63.0%)	30 (37.0%)	
Large (≥ 5 cm)	27 (100%)	17 (63.0%)	10 (37.0%)	
Metastasis				0.9703 (NS)
Negative	47 (100%)	29 (61.7%)	18 (38.3%)	
Positive	61 (100%)	39 (63.9%)	22 (36.1%)	
Clinical stage				0.2798 (NS)
I	19 (100%)	15 (78.9%)	4 (21.1%)	
II	65 (100%)	39 (60.0%)	26 (40.0%)	
III	24 (100%)	14 (58.3%)	10 (41.7%)	
ER^c^				0.1031 (NS)
Positive	51 (100%)	36 (70.6%)	15 (29.4%)	
Negative	51 (100%)	27 (52.9%)	24 (47.1%)	
Histologic grade				0.001
I	33 (100%)	28 (84.8%)	5 (15.2%)	
II	55 (100%)	33 (60.0%)	22 (40.0%)	
III	20 (100%)	7 (35.0%)	13 (65.0%)	
Nuclear grade				0.0090
1	6 (100%)	6 (100%)	0 (0%)	
2	87 (100%)	57 (65.5%)	30 (34.5%)	
3	15 (100%)	5 (33.3%)	10 (66.7%)	
Mitotic number (*n*/10 hpf^d^)				0.0016
<10	49 (100%)	37 (75.5%)	12 (24.5%)	
10–19	41 (100%)	26 (63.4%)	15 (36.6%)	
≥20	18 (100%)	5 (27.8%)	13 (72.2%)	
Ki-67 labeling index^e^				0.0078
L (<25.5)	63 (100%)	58 (76.2%)	15 (23.8%)	
H (≥ 25.5)	41 (100%)	20 (48.8%)	21 (51.2%)	
DNA ploidy^f^				0.0095
Diploidy	17 (100%)	16 (94.1%)	1 (5.9%)	
Aneuploidy	77 (100%)	44 (57.1%)	33 (42.9%)	

^a^χ^2 ^test. ^b^Not significant (*P *> 0.05). ^c^Estrogen receptor (ER): because of an insufficient amount of some carcinoma tissues, we could not obtain meaningful ER staining data from six of the 108 breast carcinoma tissues. ^d^hpf, high power field. ^e^Ki-67 (%): labeling index of Ki-67. The H and L groups were categorized in terms of being higher or lower than the mean index (25.5) in all cases. The mean indices of the L and H groups were 22.17 and 31.79, respectively. Because of an insufficient amount of some carcinoma tissues, we could not obtain meaningful Ki-67 staining data from four of the 108 breast carcinoma tissues. ^f^DNA ploidy: in some cases we did not perform flow cytometry because of insufficient tissues. In another case, we could not interpret DNA ploidy data because of a high coefficient of variance (over 8.0). We obtained meaningful data for 94 tissue samples.
